# Evaluation of In-Batch and In-Flow Synthetic Strategies towards the Stereoselective Synthesis of a Fluorinated Analogue of Retro-Thiorphan

**DOI:** 10.3390/molecules24122260

**Published:** 2019-06-18

**Authors:** Margherita Pirola, Alessandra Puglisi, Laura Raimondi, Alessandra Forni, Maurizio Benaglia

**Affiliations:** 1 Dipartimento di Chimica, Università degli Studi di Milano, Via Golgi 19, 20133 Milano, Italy; margherita.pirola@unimi.it (M.P.); alessandra.puglisi@unimi.it (A.P.); lauramaria.raimondi@unimi.it (L.R.); 2 Istituto di Scienze e Tecnologie Molecolari–ISTM-CNR, Via Golgi 19, 20133 Milano, Italy; alessandra.forni@istm.cnr.it

**Keywords:** fluorinated derivatives, flow chemistry, organocatalysis, stereoselective synthesis, reduction

## Abstract

A stereoselective synthetic strategy for the preparation of trifluoromethylamine mimics of retro-thiorphan, involving a diastereoselective, metal-free catalytic step, has been studied in batch and afforded the target molecule in good yields and high diastereoselectivity. A crucial point of the synthetic sequence was the catalytic reduction of a fluorinated enamine with trichlorosilane as reducing agent in the presence of a chiral Lewis base. The absolute configuration of the key intermediate was unambiguously assigned by X-ray analysis. The synthesis was also investigated exploiting continuous flow reactions; that is, an advanced intermediate of the target molecule was synthesized in only two in-flow synthetic modules, avoiding isolation and purifications of intermediates, leading to the isolation of the target chiral fluorinated amine in up to an 87:13 diastereoisomeric ratio.

## 1. Introduction

The unique properties of fluorine, a small and highly electronegative atom, are widely employed in medicinal chemistry to tune different aspects of the molecular properties of drugs, or drug candidates [1]. The introduction of fluorinated groups or fluorine atoms in specific positions of a molecule allows to modulate its physical chemistry, potency and pharmacokinetics [2]. In case of chiral compounds, the need to control the absolute stereochemistry of the target molecule adds a further synthetic challenge and calls for the development of novel efficient, possibly green, enantioselective catalytic methods of wide general applicability [3].

**Figure 1:**
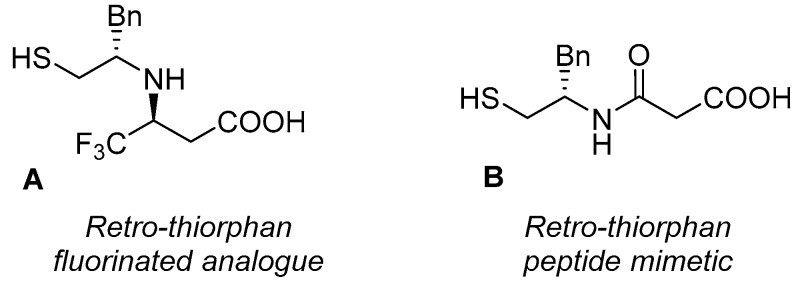
Retro-thiorphan peptide mimetic and its fluorinated analogue.

The incorporation of a trifluoromethyl group has found application also in peptides and proteins, in an attempt to improve the biological activity and modify the metabolic properties [4]. Of particular interest is the replacement of a peptide or amide bond [CONH] with trifluoroethylamine units, to provide the so called ψ [CH(CF_3_)NH] isosteres [5,6,7]. In this context, Zanda and co-workers provided a synthesis of trifluoromethylamine mimics of retro-thiorphan A (Figure 1), which was tested as an inhibitor of metalloproteinase NEP (neutral endopeptidase) [8].

**Scheme 1:**
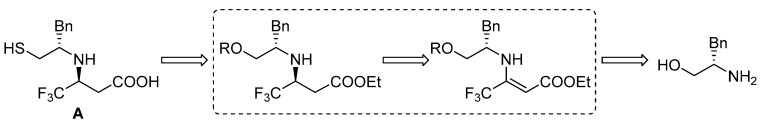
Retrosynthetic approach to **A.**

None of the two known procedures for the synthesis of this fluorinated compound was completely satisfactory: The first strategy gives a diastereomerically pure compound, but the procedure proved to be not completely reliable; the second synthetic approach, although more solid and reproducible, did not lead to the isolation of single diastereoisomers [8]. Thus, a new, more practical strategy is needed to realize a truly efficient diastereoselective synthesis of this target compound. Following our recent studies on the synthesis of chiral fluorinated molecules [9,10,11,12], the key step of our approach towards the synthesis of the fluorinated analogue of retro-thiorphan would be a diastereoselective reduction with a proper HSiCl_3_/Lewis base combination (Scheme 1). 

The employment of trichlorosilane in the presence of catalytic amounts of a chiral Lewis base is a well-established methodology to perform enantioselective reduction of carbon–nitrogen double bonds of α,β-unsaturated compounds [13,14], and represents a metal-free approach for the synthesis of pharmaceutically relevant compounds.

## 2. Results and Discussion

The synthetic strategy involves the preparation of the enamine **5**, starting from commercially available enantiomerically pure amino alcohol **1**, which needs to be protected at the hydroxyl moiety. 

### 2.1. Synthetic Strategy: In Batch Optimization

**Scheme 2:**
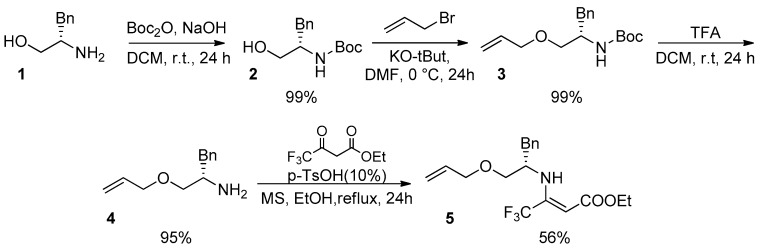
Synthetic strategy for enamine preparation.

We first reacted the *N*-Boc-protected amino alcohol **2** with allylbromide and KO-*t*Bu in *N,N*-dimethylformamide (DMF), to give the corresponding allylated compound **3** in 99% yield. Then, *N*-Boc-protected amine **3** was de-protected with trifluoroacetic acid in dichlorometane (1:1). After a basic cleavage of the ammonium salt, product **4** was obtained in 95% yield [15]. Different attempts for enamine formation were performed on ethyl 4,4,4-trifluoroacetate, to form enamine **5**. Obtained results proved that a catalytic amount of acid and ethanol as solvent are the best conditions for this condensation to afford the desired key intermediate as the 98/2 *E*/*Z* ratio (Scheme 2).

### 2.2. Catalytic Tests

**Scheme 3:**
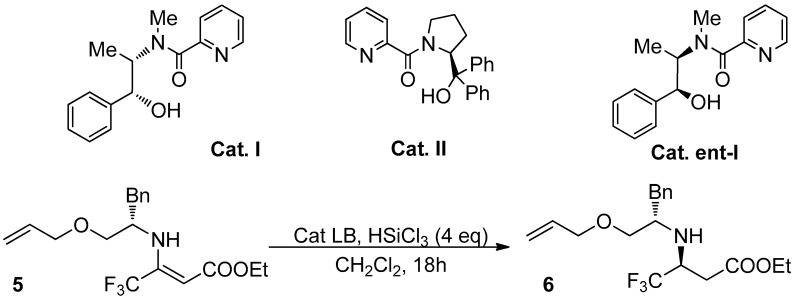
Diastereoselective catalytic reduction of enamine.

We further investigated the catalytic reduction of fluorinated enamine **5** with trichlorosilane as reducing agent in the presence of a Lewis base (Scheme 3).

First, the addition of *N,N*-dimethylformamide (DMF) as achiral Lewis Base was performed, to evaluate the simple diastereoselectivity exerted by the stereocenter of the phenylalaninol residue. The reaction afforded product **6** in 22% yield and a 67:34 diastereomeric ratio (*d.r.*) (Scheme 3 and Table 1).

Then catalyst **I**, derived from the chiral scaffold of (−)-(1*R*,2*S*)-ephedrine [16], was tested; the reduction yield was improved, but the other diastereoisomer was preferentially obtained, thus proving to be a mismatch couple with the chiral substrate (Table 1). Therefore, we decided to test catalyst **II**, known to lead to the formation of the opposite enantiomer of catalyst **I** when used in the presence of fluorinated substrates [9]. Indeed, catalyst **II** showed to be a matching combination with enamine **5** and gave slightly better results than DMF, improving both yield and the diastereomeric ratio. We then decided to synthetize the opposite enantiomer of catalyst **I**, starting from (+)-(1*S*,2*R*)-ephedrine, which proved to be a very active catalyst; that is, at a lower temperature, the *d.r.* was improved up to 95:5.

**Figure 2:**
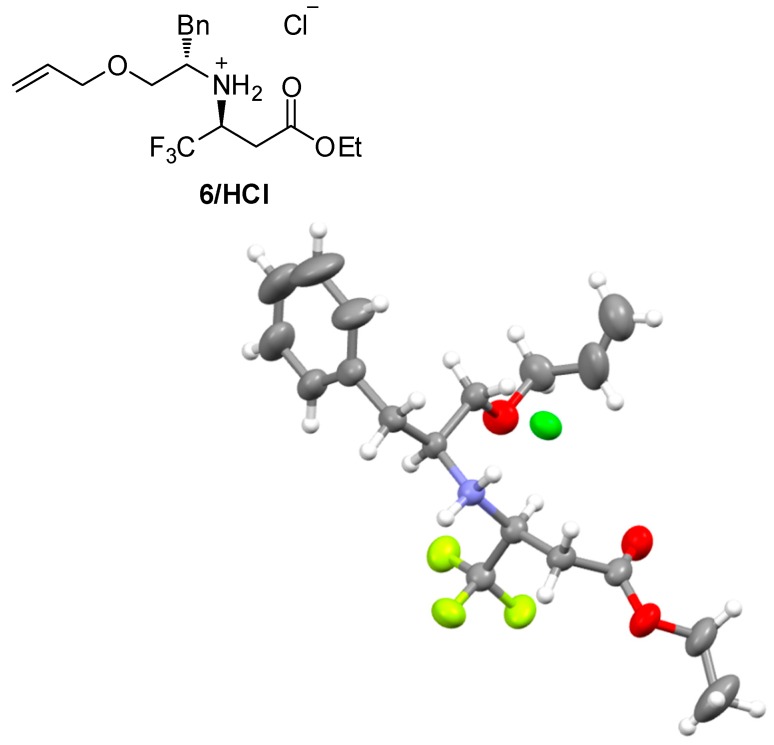
X-ray determined structure of chiral intermediate (*S,S*) **6**/HCl.

In order to confirm the hypothesized (*S,S*) absolute configuration of the key intermediate **6**, the chiral amine was treated with an equimolar amount of HCl in diethyl ether, to afford the corresponding hydrochloride salt **6**/HCl. Slow crystallization from an Et_2_O/Hexane mixture afforded light yellow crystals. X-ray analysis allowed to unambiguously determine the absolute configuration of the product to be (*S,S*), as reported in Figure 2.

### 2.3. Derivatization

**Scheme 4:**
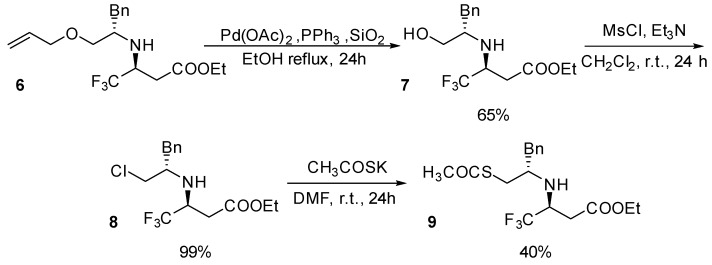
Derivatization of the fluorinated amine to the direct precursor **9** of the target product.

After the reduction, product **6** was deprotected, using a Pd(OAc)_2_/SiO_2_ catalyzed procedure in the presence of PPh_3_ in EtOH, to give the amino-alcohol **7** in 65% yield (Scheme 4).

Then, compound **7** was reacted with methanesulfonyl chloride to give the corresponding chloride **8** in 99% yield, via mesylation followed by the formation of an aziridinium cationic ring, opened by the chloride anion. Intermediate **8** was used as crude and reacted with potassium thioacetate to afford in 40% yield product **9**, the direct precursor of the final analogue [8].

### 2.4. Continuous Flow Strategy

**Scheme 5:**
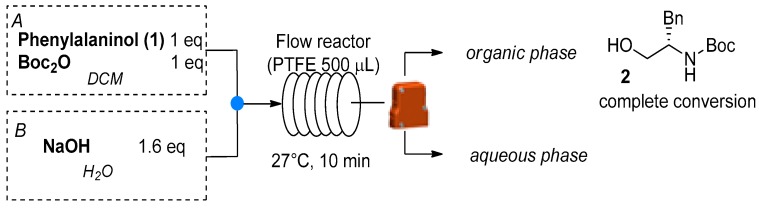
Protection of phenylalaninol performed under continuous flow in a biphasic mixture.

Considering the rising interest for the in-flow preparation of active pharmaceutical ingredients (API’s) [17,18,19], based on these results and with the aim to further accelerate the reaction, we explored the possibility of developing a continuous flow method for the preparation of the target compound. We started with the Boc-protection of phenylalaninol, performed under the same condition as in batch, with a phase separator at the end of the coil reactor. All continuous flow reactions were performed in a polytetrafluoroethylene (PTFE) coil reactor (0.58 mm internal diameter ID, and different lengths depending on the desired reactor volume—see Supporting Information). We were able to obtain the desired product with complete conversion, just by evaporating the solvent of the organic phase (Scheme 5).

**Scheme 6:**
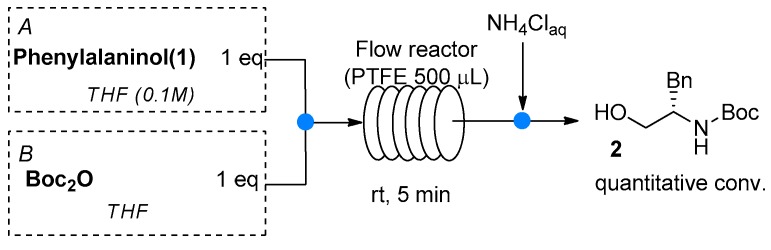
Continuous flow protection of phenylalaninol.

With the aim of developing a multistep continuous synthesis, without isolation of intermediates, since dichloromethane is not compatible with the use of potassium tert-butoxide due to possible carbene formation, we decided to switch to tetrahydrofurane (THF) as solvent for the first step. Using a more polar solvent an external base was not needed, and the desired Boc-protected aminoalcohol could be obtained in just 5 min of residence time at room temperature (Scheme 6).

**Scheme 7:**
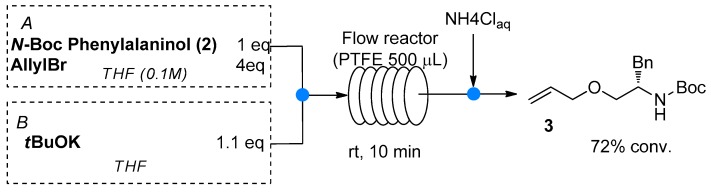
Protection of the hydroxyl moiety as allyl ether performed under continuous flow.

Then we tested the second step under continuous flow, and we were pleased to see that in only 10 min of residence time, the desired product **3** was obtained in 72% conversion (Scheme 7). We could not further optimize this reaction, due to solubility problems of by-products formed during the reaction.

**Scheme 8:**
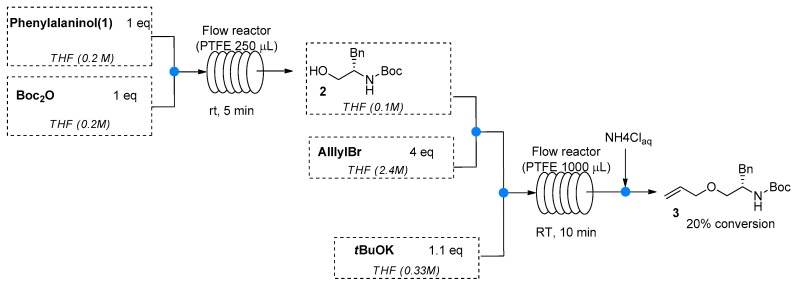
Multi-step continuous flow protection of phenylalaninol.

Unfortunately, when the two steps were combined in a single continuous flow system (Scheme 8), low conversion was observed for the second reaction, probably due to the influence of by-products formed in the first transformation.

**Scheme 9:**
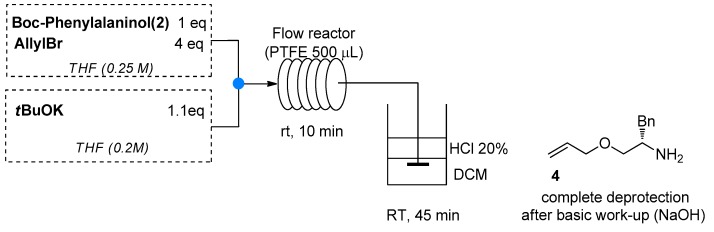
Continuous flow hydroxyl moiety protection and Boc de-protection.

Therefore, we decided to start from commercially available Boc-protected phenylalaninol **2** and synthesize the *O*-allyl protected phenyl alaninol ether. After the in-flow allylation, the reaction outcome was poured directly into a biphasic mixture of dichloromethane and aqueous HCl (20%), where it was stirred for 45 min. After an aqueous basic work-up, the desired product **4** was obtained with 75% yield, as a completely de-protected compound (Scheme 9).

**Scheme 10:**
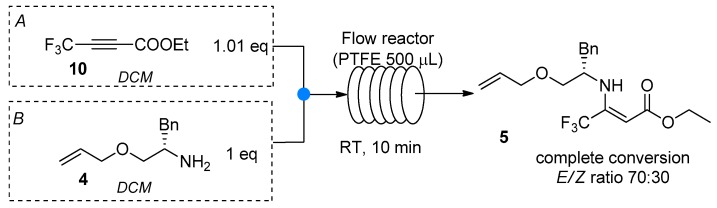
Enamine formation under continuous flow.

In order to develop a continuous flow strategy for the synthesis of enamine **5**, we decided to change the partner of our amine, choosing commercially available fluorinated alkyne **10** [20]. The reaction occurred with complete conversion in just 10 min of residence time, in dichloromethane, the same solvent of the subsequent diastereoselective reduction (Scheme 10). Then we looked at the catalytic enamine reduction mediated by trichlorosilane.

**Scheme 11:**
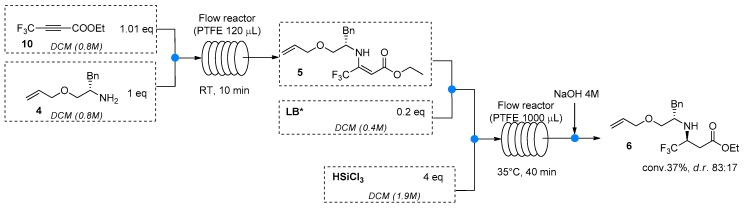
Multistep continuous flow enamine formation and reduction.

Our group already demonstrated that HSiCl_3_-mediated diastereoselective imine reduction could be efficiently performed in (micro)-mesoreactors under continuous flow conditions [21]. The reaction, performed in the coil reactor (PTFE, 1.58 mm OD, 0.58 mm ID, and tubes connected using standard HPLC connectors—see Supporting Information) afforded the expected product, although the reduction step revealed to be slower than in batch, and required long residence times (Scheme 11). To increase the yield, we heated up the reactor to 35 °C, obtaining up to 37% isolated yield, but with lower *d.r*., while cooling to room temperature, the yield was below 20%, with a 95:5 *d.r.* (see Supplementary Materials for experimental details).

## 3. Conclusions

In the present study, a highly stereoselective, metal-free strategy for the synthesis of a fluorinated chiral amine, a direct precursor of a retro-thiorphan fluorinated analogue, was developed. After setting up reaction conditions in batch, the synthesis was studied also under continuous flow conditions. In particular, two synthetic modules were set up, avoiding isolation and purification of intermediates. The desired product, an enantiomerically pure advanced precursor of the target molecule, was obtained in modest to good yields and high diastereomeric ratios, both in batch and under continuous flow. In particular, the batch synthetic strategy afforded the desired intermediate **6** in four synthetic steps with an overall yield of 31% and a *d.r.* of 95:5, starting from the commercially available Boc-protected phenylalaninol **2**, involving the stereoselective metal-free trichlorosilane-mediated enamine reduction [22,23,24,25,26,27]. Under continuous flow conditions the same intermediate **6** was obtained in only two synthetic modules (with the same number of synthetic steps), with an overall yield of 26% and a *d.r.* of 83:17. Furthermore, the Boc-protection was studied both in batch and flow conditions giving quantitative yield. The present work represents a further step towards the development of a multistep continuous flow process for the synthesis of enantiomerically pure, fluorinated, pharmaceutically relevant products.

## 4. Materials and Methods 

Reactions were monitored by analytical thin-layer chromatography (TLC) using silica gel 60 F_254_ pre-coated glass plates (0.25 mm thickness) and visualized using UV light. Flash chromatography was carried out on silica gel (230–400 mesh). Proton NMR spectra were recorded on spectrometers operating at 300 MHz (Bruker Avance 300, Bruker BioSpin, Billerica, MA, USA). Proton chemical shifts were reported in ppm (δ) with the solvent reference relative to tetramethylsilane (TMS) employed as the internal standard (CDCl_3_, δ = 7.26 ppm). ^13^C-NMR spectra were recorded on 300 MHz spectrometers (Bruker Fourier 300) operating at 75 MHz, with complete proton decoupling. Carbon chemical shifts were reported in ppm (δ) relative to TMS with the respective solvent resonance as the internal standard (CDCl_3_, δ = 77.0 ppm). ^19^F-NMR spectra were recorded on 300 MHz spectrometers (Bruker Fourier 300) operating at 282.1 MHz. Fluorine chemical shifts were reported in ppm (δ) relative to CF3Cl with the respective solvent resonance as the internal standard (CDCl_3_, δ = 77.0 ppm). Mass spectra and accurate mass analysis were carried out on a VG AUTOSPEC-M246 spectrometer (MasSpec Consulting Inc.,Oakville, ON, Canada, double-focusing magnetic sector instrument with EBE geometry) equipped with an EI source or with an LCQ Fleet ion trap mass spectrometer, ESI source, with acquisition in positive ionization mode in the mass range of 50–2000 m/z. X-ray data were collected on a Bruker Smart Apex CCD area detector (Bruker AXS Inc., Madison, WI, USA) equipped with a fine-focus sealed tube operating at 50 kV and 30 mA, using graphite-monochromated Mo Kα radiation (λ = 0.71073 Å). The fluidic device was realized by assembling coil-reactors, connected by T-junctions using standard HPLC connectors. Coil-reactors consisted of PTFE tubing (diameter: 0.58 mm) coiled in a bundle. Syringe pump: Chemix Fusion 100, equipped with two Hamilton gastight syringes.

Dry solvents were purchased and stored under nitrogen over molecular sieves (bottles with crown caps). All chemicals were purchased from commercial suppliers and used without further purification unless otherwise specified.

### 4.1. General Procedure for the Stereoselective Catalytic Reduction of Enamine 5 under Batch Conditions

Dry DMF or the appropriate catalytic chiral Lewis base in the reported amount (Table 1), and a 0.1 M solution of enamine **5** (1 equiv.) in dry CH_2_Cl_2_ were introduced in a round bottomed flask under nitrogen atmosphere. The mixture was cooled down to the indicated temperature and HSiCl_3_ (3.5 equiv.) was added to the reaction mixture. After the desired time, the reaction was quenched with a 4 M solution of NaOH until basic pH was reached. The resulting mixture was extracted with CH_2_Cl_2_, separated, and the organic phase dried over anhydrous Na_2_SO_4_. The solvent was removed under reduced pressure and the residue purified by column chromatography (silica gel, hexanes/EtOAc = 98:2) to afford **6** as a colorless oil. 

### 4.2. General Procedure for the Multistep Formation and Stereoselective Catalytic Reduction of Enamine 5 under Flow Conditions

Two 2.5 mL Hamilton gastight syringes, one containing compound **10** (2 mL of a 0.8 M solution in CH_2_Cl_2,_ 1.03 equiv.) and the other compound **4** (2 mL of a 0.8 M solution in CH_2_Cl_2_, 1 equiv.) were connected by a PEEK tee junction to a 250 µL PTFE coil reactor. Both syringes fed the solutions at 6 µL/min, giving a residence time of 10 min. The outcome of the reactor was connected to another tee junction, fed by a 1 mL Hamilton gastight syringe, containing cat. **ent-I** (0.8 mL of 0.4 M solution in CH_2_Cl_2_, 0.2 equiv.), feeding at 2.5 µL/min. The outcome of this second tee was connected to another tee junction, fed by a 5 mL Hamilton gastight syringe, containing HSiCl_3_ (3.3 mL of a 1.9 M solution in CH_2_Cl_2,_ 4 equiv.) with a flow rate of 10 µL/min. The outcome of this third tee was connected to a 1000 µL PTFE coil reactor, with a total flow rate of 24.5 µL/min, and a subsequent residence time of 40 min. The outcome of the reactor was collected into a NaOH 4 M solution at 0 °C. After the first two volumes were discharged, steady state conditions were reached. The conversion was reported as an average of three reactors volume, separately collected, and confirmed as isolated yield.

## Figures and Tables

**Table 1:** Diastereoselective catalytic reductions.

Entry	Lewis Base	Cat. eq	T (°C)	*d.r.* ^1^	Yield (%) ^2^
1 ^3^	DMF	5	0	67:34	22
2	Cat. **I**	0.2	0 to rt	34:66	40
3	Cat. **II**	0.2	0 to rt	81:19	38
4	Cat. ent-**I**	0.2	0 to rt	83:17	70
5	Cat. ent-**I**	0.2	-10	95:5	60
6	Cat. ent-**I**	0.1	-10	95:5	53
7	Cat. ent-**I**	0.1	-20	96:4	37

^1^ Calculated by ^19^F-NMR on the crude mixture. ^2^ Isolated yield. ^3^ Reaction time 48 h.

## Supplementary Materials

The supplementary materials are available online.
