# Supplementary material for: Evaluation of In-Batch and In-Flow Synthetic Strategies towards the Stereoselective Synthesis of a Fluorinated Analogue of Retro-Thiorphan
Source: Molecules. 2019 Jun 18;24(12):2260. doi: 10.3390/molecules24122260 (PMC6631789; doi:10.3390/molecules24122260)

Evaluation of in-batch and in-flow synthetic strategies towards the stereoselective synthesis of a fluorinated analogue of retro-thiorphan.

M. Pirola,[a] A. Puglisi,[a] L. Raimondi,[a] A. Forni,[b] M. Benaglia[a]

[a] Dipartimento di Chimica, Università degli Studi di Milano, via Golgi 19, 20133, Milan, IT, Italy

[b] Istituto di Scienze e Tecnologie Molecolari – ISTM-CNR, Via Golgi 19, 20133 Milano

Supporting Information

Table of contents

Table of contents S1

Catalysts synthesis S2

In batch synthetic strategy S3

In flow synthetic strategy S14

Pictures of flow reactors S19

Catalysts synthesis

General procedure for the synthesis of catalysts I, II and ent-I.[1]

A solution of ethyl chloroformate (1 mmol, 1 equiv.) in dry THF (2 mL) was dropped in a solution of 2-picolinic acid (1 mmol, 1 equiv.) and TEA (1 mmol, 1 equiv.) in dry THF (25 mL) at 0 °C within 15 min. The resulting mixture was stirred for 1 h before 1 mmol (1 equiv.) of the proper amino alcohol in dry THF (10 mL) was added dropwise. The mixture was stirred at 0°C for 1 h and then at room temperature for overnight. The mixture was quenched with water. The aqueous layer was extracted with ethyl acetate (3 × 15 mL) and the combined organic layer was dried over $MgSO_4$ for 2 h, filtered and concentrated under reduced pressure.

Cat I and Cat ent-I

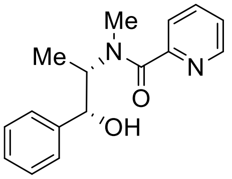

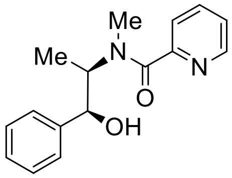

Purified by flash column chromatography. White solid, yield 73%.
Rf = 0.66 in DCM/MeOH 95:5
Rotamer 1. ^{1}H NMR (300 MHz, $CDCl_3$): δ 7.4–7.1 (m, 10H), 6.98 (s, 1H), 4.81 (s, 1H), 4.5 (br s, 1H), 2.6 (s, 3H), 1.33 (d, 3H).
Rotamer 2. ^{1}H NMR (300 MHz, $CDCl_3$): δ 7.4–7.1 (m, 10H), 6.90 (s, 1H), 4.5 (s, 1H), 4.3 (br s, 1H), 2.9 (s, 3H),1.4 (d, 3H).

Cat II

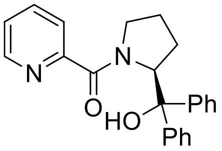

Purified by flash column chromatography. White solid, yield 43%.
Rf = 0.14 in hexane/EtOAc 7:3

[1] Y. Matsumura, K. Ogura, Y. Kouchi, F. Iwasaki, O. Onomura; *Org. Lett.;* **2006**, *17*, 3789.

^{1}H NMR (300 MHz, $CDCl_3$) δ 8.56 and 8.26 (2×br s, 1H), 7.71-7.77 (m, 1H), 7.15-7.52 (m, 12H), 6.69 (s, 1H), 5.42-5.50 (m, 1H), 3.19-3.97 (m, 2H), 2.03-2.14 (m, 2H), 1.50-1.82 (m, 1H), 0.75-1.25 (m, 1H).

In batch synthesis of the target molecule

Synthesis of *(S)*-tert-butyl (1-hydroxy-3-phenylpropan-2-yl)carbamate[2] **2**

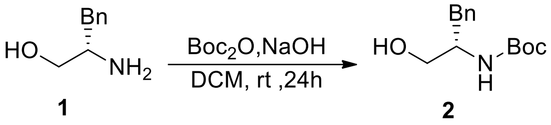

A solution of tert-butyl dicarbonate (1eq, 6.7mmol) in DCM (14 mL) was added dropwise to a solution of *(S)-2-amino-3-phenylpropan-1-ol* (**1**) (1eq, 6.7mmol) in a mixture of DCM (14 mL) and 1N NaOH (11 mL). The reaction mixture was then stirred at room temperature for 24 h, and the organic phase was separated. The aqueous layer was extracted with DCM (10 mL x 2). The combined organic layers were washed with water (10 mL x 1) and dried over Na_2SO_4. The solvent was evaporated in vacuo, to afford *(S)-tert-butyl (1-hydroxy-3-phenylpropan-2-yl)carbamate* (**2**) as a white solid; the crude has been used without further purification in the following synthetic cascade. All analytical data are in agreement with literature.

Yield = 99%

Rf = 0.1 in hexanes/EtOAc 7:3

^{1}H NMR (300 MHz, $CDCl_3$) δ 7.32 – 7.21 (m, 5H); 4.72 (br d, 1H); 3.89 (m, 1H); 3.67 - 3.55 (m, 2H); 2.86 (d, 2H); 1.43 (s; 9H).

Synthesis of (*S*)-tert-butyl (1-(allyloxy)-3-phenylpropan-2-yl)carbamate[2] **3**

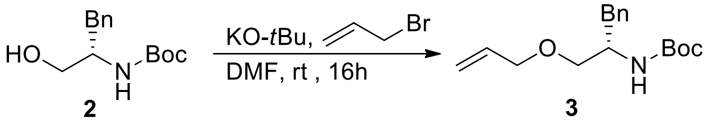

A solution of (*S*)-tert-butyl (1-hydroxy-3-phenylpropan-2-yl)carbamate (**2**) (1 eq, 6.7mmol), 4eq of allylbromide and DMF (0.1 M) was prepared in a flask and stirred under ice bath for 15 min. 1.1eq of KO-*t*Bu were added to the solution portion wise. The thus obtained mixture was stirred for 4h at 0°C and

[2] Jogula, S.; Dasari, B.; Khatravath, M.; Chandrasekar, G.; Kitambi, S. S.; Arya; P. *Eur. J. Org. Chem*. **2013**, *23*, 5036-5040.

for 12h at r.t. The mixture was diluted with EtOAc and treated with HCl 10%, then the organic phase was treated with $NaHCO_3$ (ss), and finally washed with brine. The organic phases were reunited, dried over Na_2SO_4, filtered, and then concentrated under vacuum at high temperature to give a crude colourless oil which was used without further purification in the following synthetic step.

All analytical data are in agreement with literature.

Yield = 99%

Rf = 0.55 in hexane/EtOAc 7:3

^{1}H NMR (300 MHz, $CDCl_3$) δ 7.32 – 7.22 (m, 5H), 5.98 – 5.87 (m, 1H), 5.30 - 5.19 (m, 2H), 4.86 -4.74 (br, 1H), 3.98 – 3.88 (m, 3H), 3.36 (m, 2H), 2.80 (dd, 2H), 1.44 (s, 9H).

Synthesis of (*S*)-1-(allyloxy)-3-phenylpropan-2-amine[2] **4**

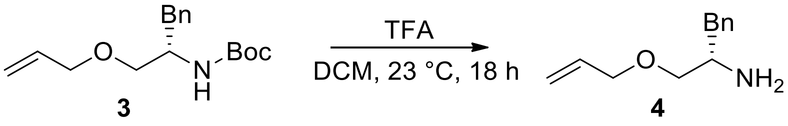

To the *O*-allyl amine **3** (0.39 mmol, 1 eq.) in 10 mL of dry CH_2Cl_2, TFA (3.94 mmol, 10 eq.) was added at 0 °C. Then the solution was stirred at r.t. for 18 h. After completion of the reaction (monitored by TLC), the excess of TFA was evaporated in vacuo. 10 mL of Na_2CO_3 (10 % aqueous solution) were added cautiously and the mixture was extracted with CH_2Cl_2 (2 × 10 mL). The combined organic layers were dried over anhydrous Na_2SO_4, filtered and concentrated to give a yellow oil. The crude product has been used in the next step without further purification. All analytical data are in agreement with literature.

Yield = 99%

Rf = 0.04 in hexanes/EtOAc 7:3

^{1}H NMR (300 MHz, $CDCl_3$) δ 7.31 – 7.06 (m, 5H), 5.98 – 5.72 (m, 1H), 5.31 – 5.04 (m, 2H), 3.92 (d, J = 5.6 Hz, 2H), 3.44 – 3.29 (m, 1H), 3.28 – 3.09 (m, 2H), 2.72 (dd, J = 13.3, 4.9 Hz, 1H), 2.56 – 2.41 (m, 1H).

Synthesis of (*S*,*Z*)-ethyl 3-((1-(allyloxy)-3-phenylpropan-2-yl)amino)-4,4,4-trifluorobut-2-enoate **5**

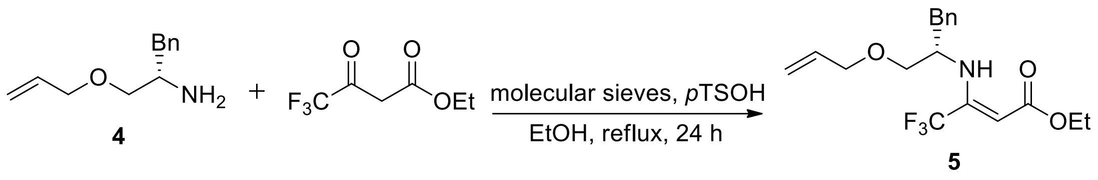

O-allyl amine **4** (0.39 mmol,1 eq.) and ethyl 4,4,4-trifluoro-3-oxobutanoate (0.39 mmol, 1 eq.) were charged in a round bottomed flask under inert atmosphere and 4 Å molecular sieves and 10 mol% of p-toluensulfonic acid were added. Then dry EtOH (3.9 mL) was added, and the reaction was heated to reflux and stirred for 24 h. The mixture was diluted with DCM, and filtrated over celite. After solvent removal, the crude was purified by column chromatography (silica gel, hexanes/EtOAc = 8:2) to afford **5** as a colorless oil.

Yield = 56%
Rf = 0.65 in hexanes/AcOEt 7:3

^{1}H NMR (300 MHz, $CDCl_3$) δ 8.36 (d, *J* = 10.5 Hz, 1H), 7.17 (dd, *J* = 15.5, 12.7 Hz, 5H), 5.97 – 5.72 (m, 1H), 5.16 (dd, *J* = 31.3, 13.8 Hz, 2H), 4.98 (s, 1H), 4.09 (d, *J* = 7.1 Hz, 2H), 4.01 – 3.88 (m, 2H), 3.72 (s, 1H), 3.33 – 3.20 (m, 2H), 2.84 (dd, *J* = 32.6, 7.0 Hz, 2H), 1.25 – 1.15 (m, 3H).
^{19}F NMR (282 MHz, $CDCl_3$) δ -66.12 (s).
^{13}C NMR (75 MHz, $CDCl_3$) δ 169.6 (s), 147.5 (q), 137.6 (s), 129.4 (s), 128.4 (s), 126.6 (s), 122.2 (q), 117.0 (s), 85.1 (q), 72.2 (s), 70.4 (s), 59.6 (s), 56.0 (s), 39.4 (s), 14.3 (s).

MS mass (ESI): 357.78 (M+)
During high resolution EI mass analysis the benzyl portion is lost:
HRMS (EI): calcd for C11H15F3O3N1: 266.100403, found: 266.100100

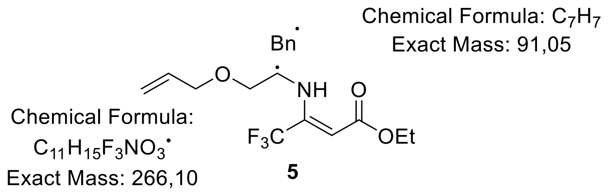

$[\alpha]^{20}_D$= -207.81 (c=1.14 g/100 mL, $CHCl_3$, λ=546 nm).

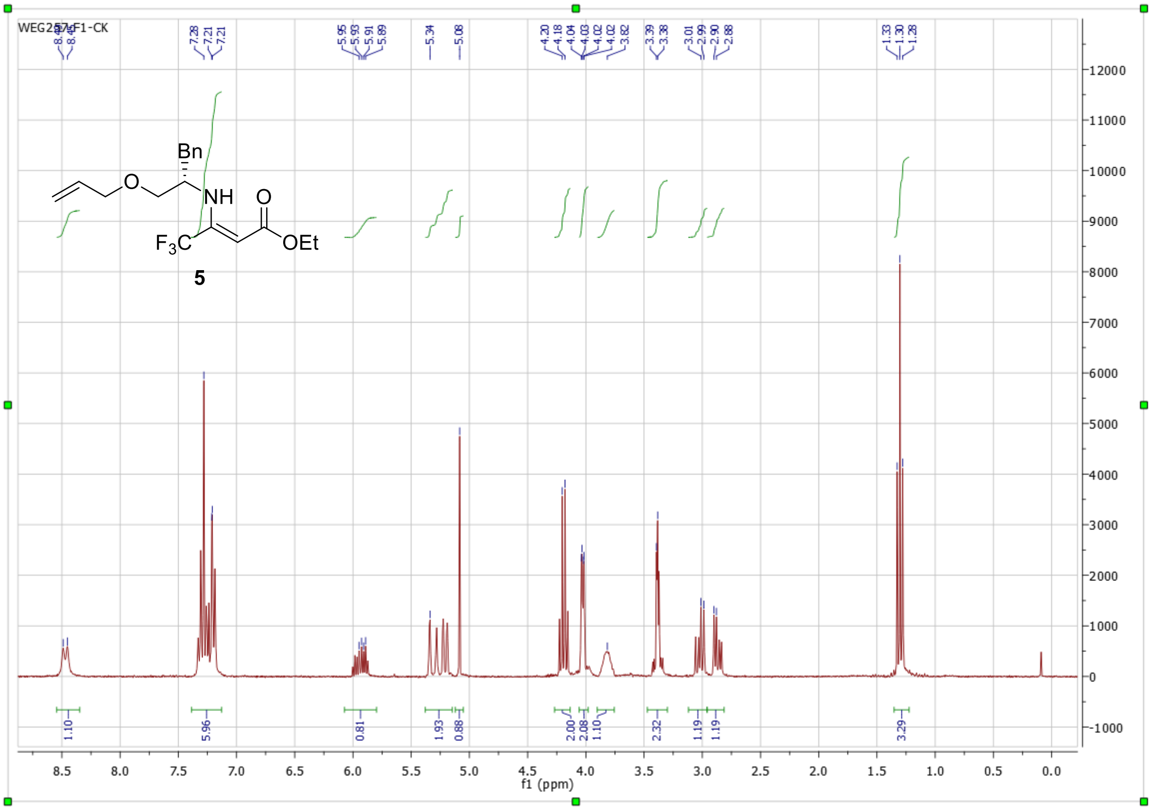
WEG257-F1-CK
Bn
NH
O
OEt
F3C
5
f1 (ppm)

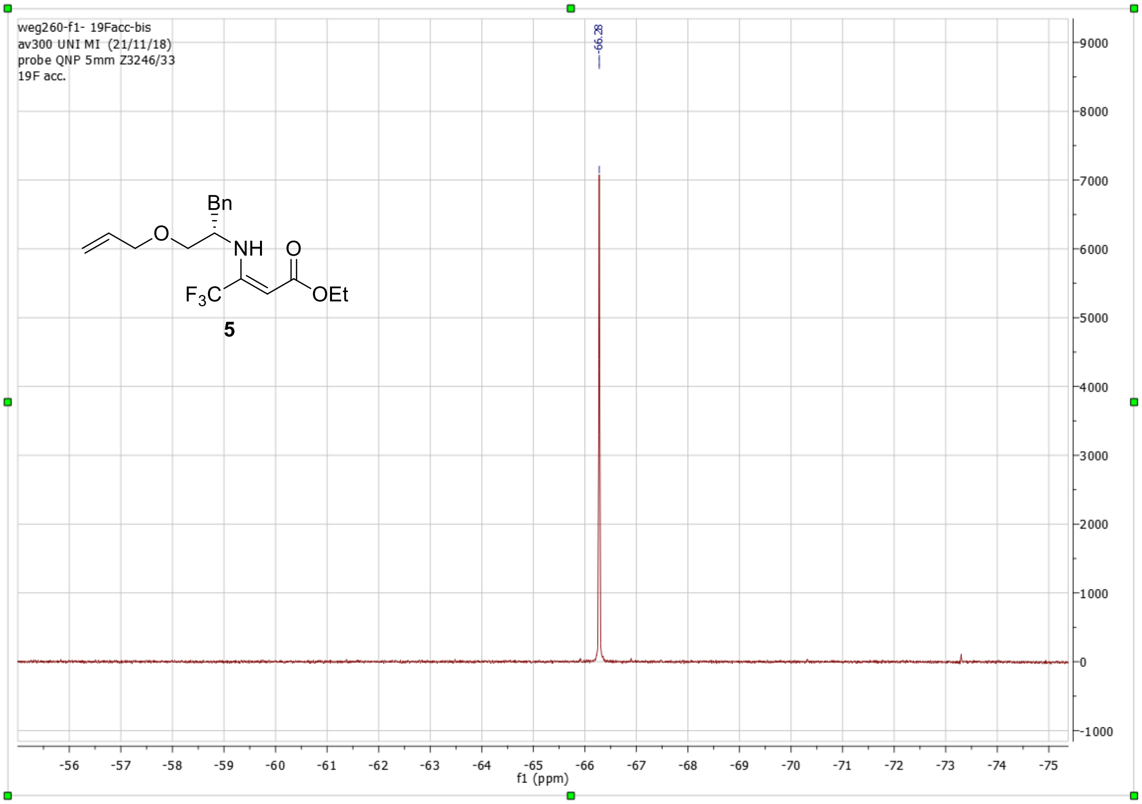
weg260-f1- 19Facc-bis
av300 UNI MI (21/11/18)
probe QNP 5mm Z3246/33
19F acc.
-66.28
Bn
NH
O
OEt
F3C
5
f1 (ppm)

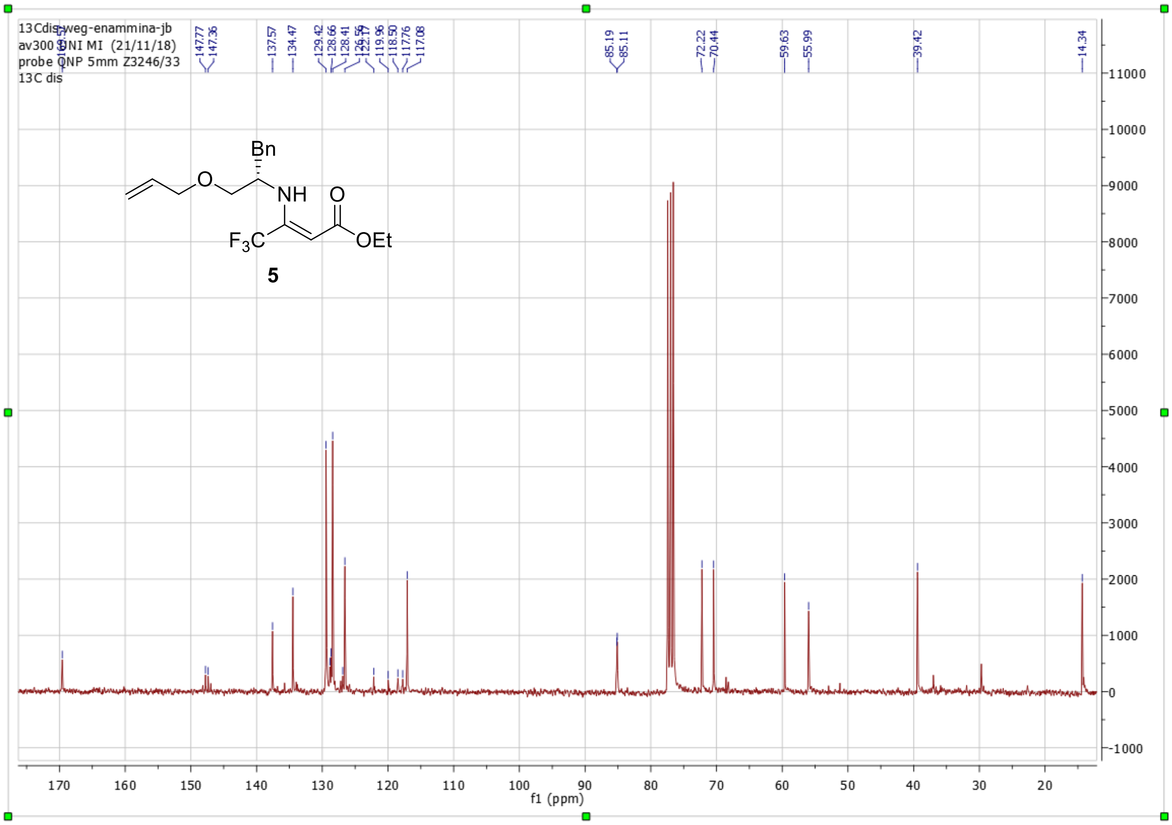

Synthesis of Ethyl 3-(((*S*)-1-(allyloxy)-3-phenylpropan-2-yl)amino)-4,4,4-trifluorobutanoate **6**

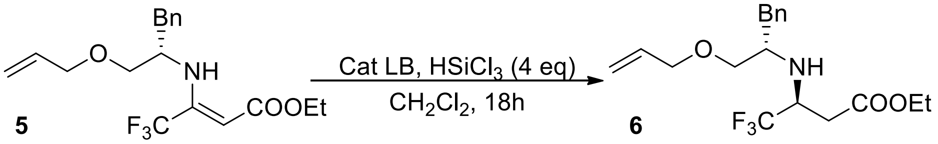

Dry DMF or the appropriate catalytic chiral Lewis Base in the reported amount (Table 1), and a 0.1 M solution of enamine **5** (1 eq.) in dry CH_2Cl_2 were introduced in a round bottomed flask under nitrogen atmosphere. The mixture was cooled down to the indicated temperature and $HSiCl_3$ (3.5 eq.) was added to the reaction mixture. After the desired time, the reaction was quenched with a 4 M solution of NaOH until basic pH was reached. The resulting mixture was extracted with CH_2Cl_2, separated, and the organic phase dried over anhydrous Na_2SO_4. The solvent was removed under reduced pressure. The residue was purified by column chromatography (silica gel, hexanes/EtOAc = 98:2) to afford **6** as a colorless oil.

Rf = 0.8 in hexanes/AcOEt 9:1

^{1}H NMR (300 MHz, $CDCl_3$) δ 7.31-7.10 (m, 5H), 5.98 – 5.84 (m, 1H), 5.29 - 5.16 (m, 2H), 4.18 (q, 2H), 3.95 (d, *J* = 7.1 Hz, 2H), 3.80 (br s, 1H), 3.34 – 3.20 (m, 3H), 2.78 – 2.74 (m, 2H), 2.68 (dd, 1H), 2.45

(dd, 1H), 1.29 (t, 3H).

^{19}F NMR (282 MHz, $CDCl_3$) δ -75.32 (d) *minor diast.;* -75.90 (d) *major diast*.

^{13}C NMR (75 MHz, $CDCl_3$) δ 170.0 (s), 138.3 (s), 134.7 (s), 129.3 (s), 128.5 (q), 128.4 (s), 126.3 (s), 116.7 (s), 72.0 (s), 61.0 (s), 57.2 (s), 54.8 (q), 39.0 (s), 35.47 (s), 14.1 (s).

MS mass (ESI): 382.12 (M + 23).

During high resolution EI mass analysis the allylic portion is lost:

HRMS (EI): calcd for C14H17F3O2N1: 288.121139, found: 288.121790

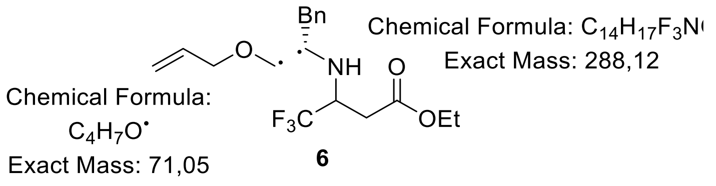

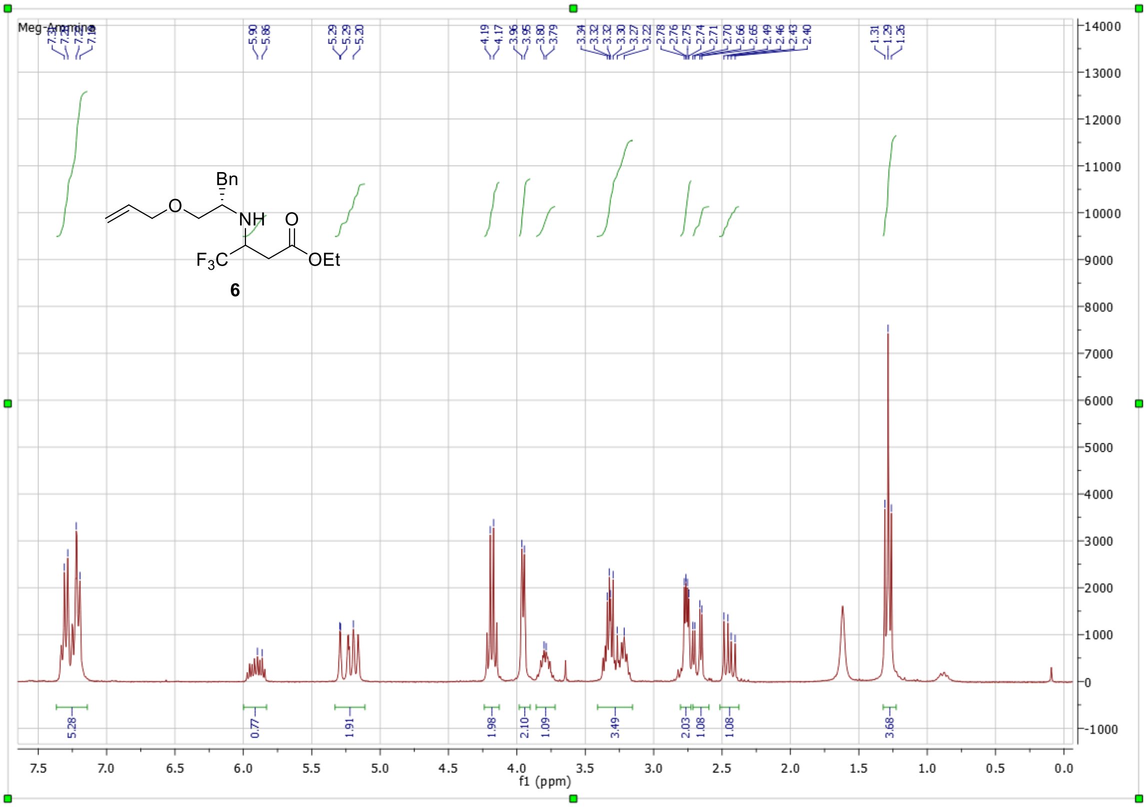

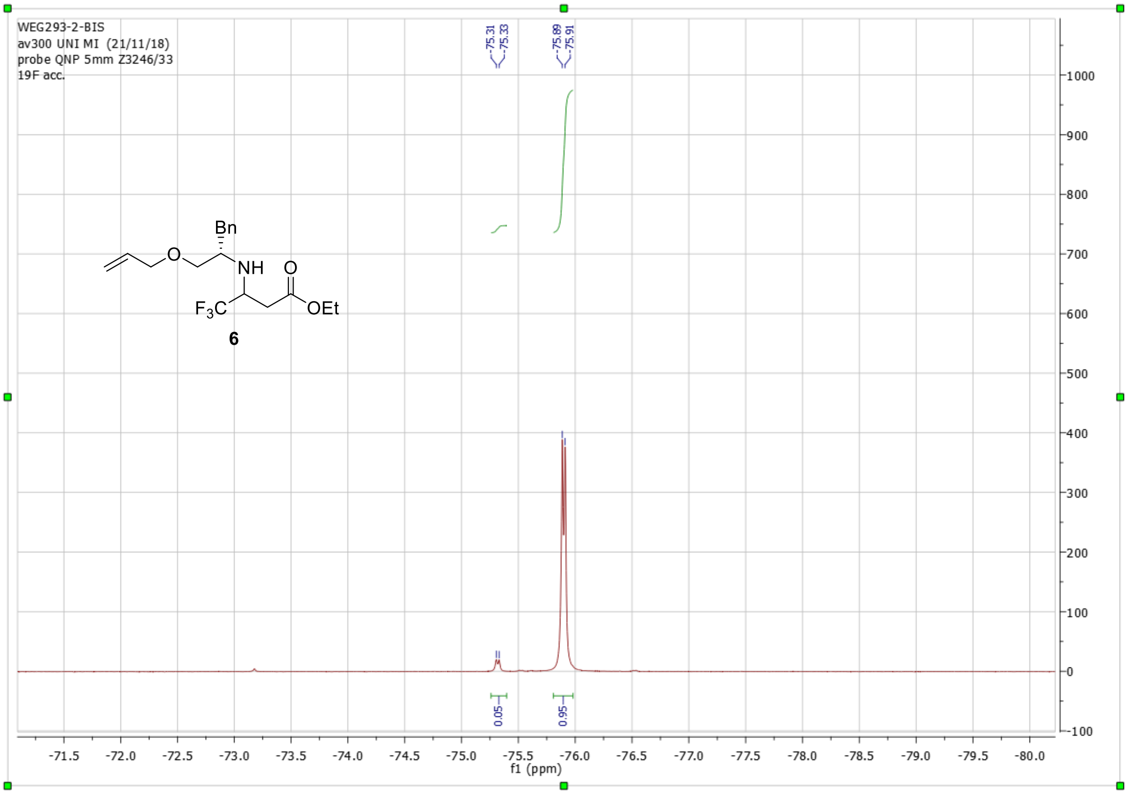
WEG293-2-BIS
av300 UNI MI (21/11/18)
probe QNP 5mm Z3246/33
19F acc.
-75.31
-75.33
-75.89
-75.91
Bn
O
NH
O
F_3C
OEt
6
0.05
0.95
f1 (ppm)

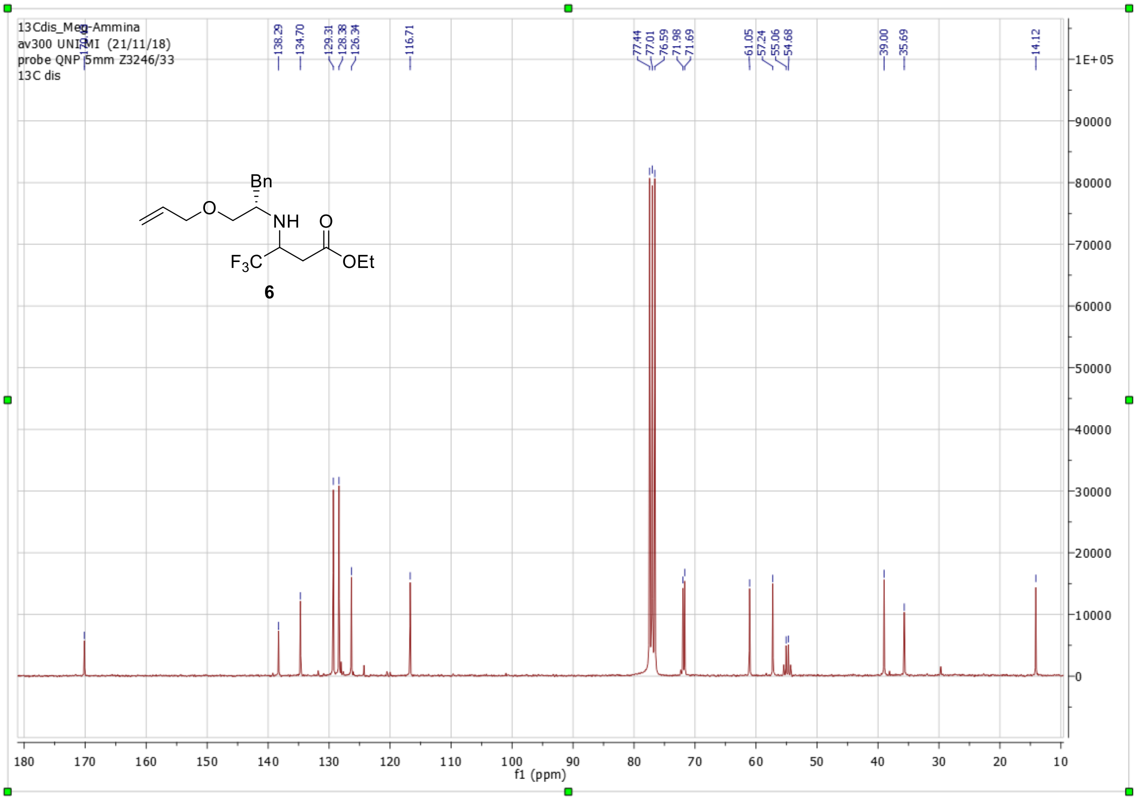
13Cdis_Meg-Ammina
av300 UNI MI (21/11/18)
probe QNP 5mm Z3246/33
13C dis
138.29
134.70
129.31
128.38
126.34
116.71
77.44
77.01
76.59
71.98
71.69
61.05
57.24
55.06
54.68
39.00
35.69
14.12
Bn
O
NH
O
F_3C
OEt
6
f1 (ppm)

In order to perform X-ray analysis, a sample of compound **6** was treated with an equimolar amount of HCl in Et_2O. The product was slowly crystallized from a mixture of Et_2O/hexane to afford light-yellow crystals of **6**/HCl. Absolute configuration was determined to be (*S*,*S*).

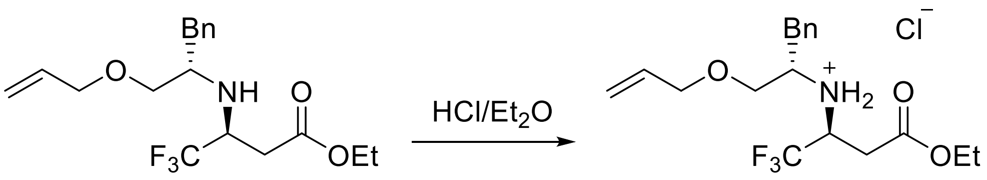

6 **6/HCl**

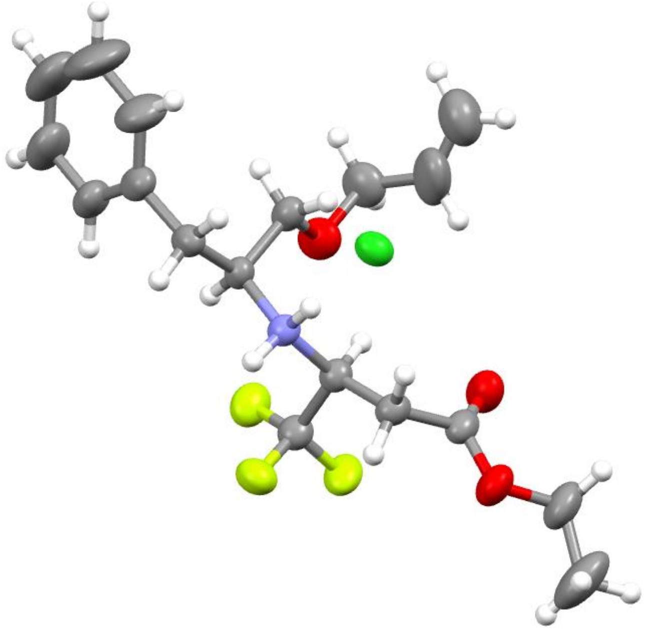

A summary of the experimental details concerning the single-crystal X-ray diffraction study of **6/HCl** is reported in Table S1. X-ray data were collected on a Bruker Smart Apex CCD area detector equipped with fine-focus sealed tube operating at 50 kV and 30 mA, using graphite-monochromated Mo Kα radiation (λ = 0.71073 Å). Data reduction was made using SAINT programs;[3] absorption corrections based on multiscan were obtained by SADABS.[3] The structures were solved by SHELXS-97[4] and refined on F2 by full-matrix least-squares using SHELXL-14.[5] The program ORTEP-III[6] was used for molecular graphics.

---

[3] Bruker, SMART, SAINT and SADABS; Bruker AXS Inc.: Madison, Wisconsin, USA, 1997.

[4] Sheldrick, G.M. A short history of SHELX. Acta Cryst. 2008, A64, 112–122.

[5] Sheldrick, G.M. Crystal structure refinement with SHELXL. Acta Cryst. 2015, C71, 3–8.

[6] M. N. Burnett, C. K. Johnson, ORTEP-III: Oak Ridge Thermal Ellipsoid Plot Program for Crystal Structure Illustrations, Oak Ridge National Laboratory Report ORNL-6895, 1996.

Table S1. Crystallographic data, data collection details and results of refinement for 6/HCl

formula, M_r	$(C_{18}H_{25}F_3NO_3)Cl$, 395.84
crystal system	Monoclinic
space group, Z	$P2_1$, 2
D_{calc}, g cm^{-3}	1.300
a, Å	12.6047(15)
b, Å	6.5793(8)
c, Å	12.8724(15)
β, °	108.694(2)
V, Å^3	1011.2(2)
crystal size, mm	0.52×0.05×0.03
color, habit	colorless, needle
μ, mm^{-1}	0.232
radiation	MoK_α
T, K	180(2)
$2\theta_{max}$, °	52.77
h, k, l ranges	-15→15; -8→8; -16→16
intensity decay, %	0.00
adsorption correction	multi-scan
T_{min}, T_{max}	0.691, 0.745
measured reflections	15272
R_{int}	0.0535
independent reflections	4126
reflections with $I>2\sigma(I)$	3015
no. of parameters	236
R, wR $[F^2>2\sigma(F^2)]$	0.0481, 0.0995
Flack parameter	-0.02(4)
goodness of fit	0.991

$\Delta\rho_{max}$, $\Delta\rho_{min}$ (eÅ^{-3})	0.356, -0.165

Tables of atomic coordinates, anisotropic thermal parameters, bond lengths and angles of $C_{18}H_{24}F_3NO_3$ may be obtained free of charge from The Director CCDC, 12 Union Road, Cambridge CB2 1 EZ, UK, on quoting the deposition numbers CCDC 1914889 the names of the authors and the journal citation (fax: +44-1223-336-033; e-mail: deposit@ccdc.cam.ac.uk; web site: http://www.ccdc.cam.ac.uk).

Synthesis of ethyl 4,4,4-trifluoro-3-(((S)-1-hydroxy-3-phenylpropan-2-yl)amino)butanoate **7**

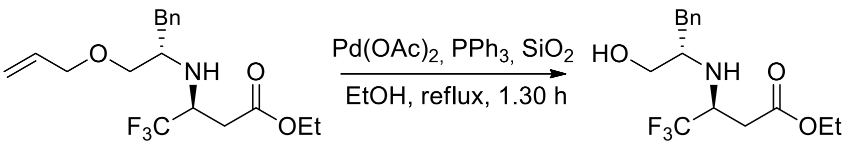

6 **7**

Compound **6** (0.13 mmol, 1 equiv.) in EtOH (1.5 mL) was introduced in a two necks round-bottomed flask equipped with a condenser under N_2 atmosphere. $Pd(OAc)_2$ (0.03 mmol, 0.2 equiv.) and PPh_3 (0.11 mmol, 0.88 equiv.) in EtOH (1.5 mL) were added to the solution, followed by 300 mg of SiO_2. The reaction mixture was stirred at reflux for 20 h. After completion of reaction (monitored by TLC), the reaction mixture was diluted with DCM and filtered over celite. After solvent removal, the crude was purified by column chromatography (silica gel, hexane/EtOAc = 9:1) to afford **7** as a yellow oil. All analytical data are in agreement with literature.[7]

Yield = 65%

Rf = 0.32 in hexane/EtOAc 7:3

^{1}H NMR (300 MHz, $CDCl_3$) δ 7.33 – 7.20 (m, 5H), 4.25 (q, 2H), 3.75 (d, 2H), 3.37 (m, 1H), 3.23 (m, 1H), 3.20 (br s, H), 2.76 – 2.71 (m, 3H), 2.44 (m, 1H), 1.22 (t, 3H).

^{19}F NMR (300 MHz, $CDCl_3$) δ -75.12 (d), -75.99 (d).

Synthesis of *(S)*-ethyl 3-(((*S*)-1-chloro-3-phenylpropan-2-yl)amino)-4,4,4-trifluorobutanoate **8**[3]

[7] Molteni, M.; Volonterio, A.; Fossati, G.; Lazzari, P.; Zanda, M. Conjugated additions of amines and β-amino alcohols to trifluorocrotonic acid derivatives: synthesis of ψ[NHCH(CF_3)]-retro-thiorphan *Tetrahedron Lett.* **2007**, 48, 589-593

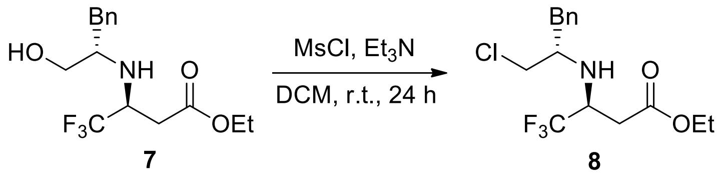

Dry Et_3N (0.31 mmol, 3 eq) and MsCl (0.31 mmol, 3 equiv.) were consecutevely added to a solution of **7** (0.10 mmol, 1 eq) in dry CH_2Cl_2 (0.1 M solution) at room temperature under N_2. After 24 h the mixture was diluted with water and extracted with CH_2Cl_2. The collected organic layers were dried (Na_2SO_4), filtered, concentrated under reduced pressure, to afford **8** as a yellow oil. The product was used without further purification, all analytical data are in agreement with literature.

Yield = 99%
Rf = 0.74 in hexanes/EtOAc 7:3

^{1}H NMR (300 MHz, $CDCl_3$) δ 7.45-6.97 (m, 5H), 4.11 (d, 2H), 3.60 (s, 1H), 3.40 (m, 2H), 3.19 (d,1H), 2.88 – 2.46 (m, 3H), 2.35 (dd, 1H), 1.20 (t,3H).

Synthesis of (S)-ethyl 3-(((S)-1-(acetylthio)-3-phenylpropan-2-yl)amino)-4,4,4-trifluorobutanoate **9**[3]

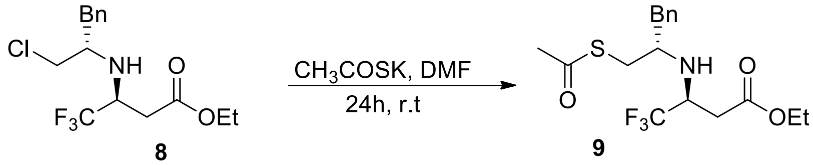

A solution of dry DMF (0.5 mL) and crude **8** (0.22 mmol, 1eq) were subsequently added to a solution of CH_3COSK (0.22 mmol, 1 eq) in dry DMF (0.5 mL) at 0ºC under N_2. After 24h at room temperature, the mixture was diluted with water and extracted with AcOEt. The collected organic layers were dried (Na_2SO_4), filtered, concentrated under reduced pressure, the crude was purified by column chromatography (silica gel, n-hexane/AcOEt from 9:1 to 7:3) to afford **9** as a brown-yellow oil.

Yield = 40%
^{1}H NMR (300 MHz, $CDCl_3$) δ 7.34-7.18 (m, 5H), 4.25-4.16 (m, 2H), 3.60 (s, 1H), 3.28-3.17 (m, 2H), 2.87-2.81 (m, 2H), 2.68-2.59 (m, 2H), 2.38 (s, 3H), 1.55-1.32 (m, 1H), 1.32-1.28 (t, 3H).

In flow synthesis of the target molecule

In flow synthesis of (*S*)-tert-butyl (1-hydroxy-3-phenylpropan-2-yl)carbamate **2**

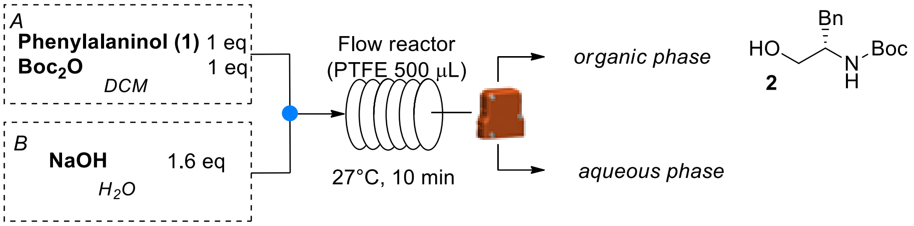

Two 2.5 mL Hamilton gastight syringes, containing *A* compound **1** (1 eq.) and Boc_2O (1 eq.) in 2 mL of CH_2Cl_2 (0.25 M), and *B* aqueous NaOH (2 mL of a 1N solution in water) were connected by a PEEK tee junction to a 500 µL PTFE coil reactor. Both syringes fed the solutions ad 25 µL/min, giving a residence time of 10 minutes. The outcome of the reactor was connected to a *Zaiput liquid liquid phase separator*, to separate the organic phase from the aqueous phase. The organic phase was dried over sodium sulphate and evaporated under vacuo, to give pure product **2**, as evaluated by ^{1}H NMR, in quantitative yield.

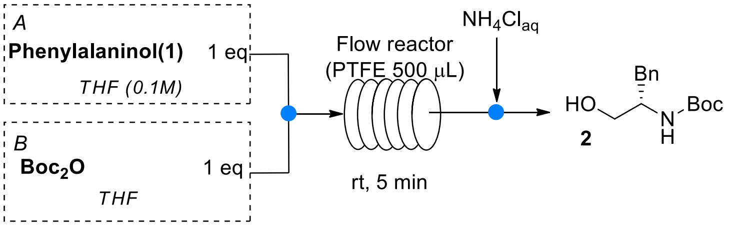

Two 2.5 mL Hamilton gastight syringes, containing *A* compound **1** (1 eq.) in 2 mL of THF (0.2 M), and *B* Boc_2O (1 eq.) in 2 mL of THF were connected by a PEEK tee junction to a 500 µL PTFE coil reactor. Both syringes fed the solutions ad 50 µL/min, giving a residence time of 5 minutes. The outcome of the reactor was collected into a vial containing saturated NH_4Cl. The organic phase was separated, dried over sodium sulphate and evaporated under vacuo, to give pure product **2**, as evaluated by ^{1}H NMR, in quantitative yield.

Analytical data are in agreement with product obtained using batch procedure.

^{1}H NMR (300 MHz, $CDCl_3$) δ 7.32 – 7.21 (m, 5H); 4.72 (br d, 1H); 3.89 (m, 1H); 3.67 - 3.55 (m, 2H); 2.86 (d, 2H); 1.43 (s; 9H).

In flow synthesis of (*S*)-tert-butyl (1-(allyloxy)-3-phenylpropan-2-yl)carbamate **3**

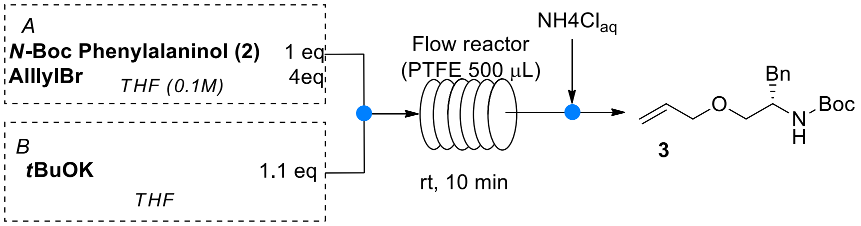

Two 2.5 mL Hamilton gastight syringes, containing *A* compound **2** (1 eq.) in 2 mL of THF (0.1 M), and 4 eq. of allylbromide, and *B* *t*BuOK (1.1 eq.) in 2 mL of THF were connected by a PEEK tee junction to a 500 µL PTFE coil reactor. Both syringes fed the solutions ad 25 µL/min, giving a residence time of 10 minutes. The outcome of the reactor was collected into a vial containing saturated NH_4Cl. The organic phase was separated, dried over sodium sulphate and evaporated under vacuo, to give product **3** with a 75% conversion, evaluated by ^{1}H NMR, and confirmed as isolated yield.

Analytical data are in agreement with product obtained in batch procedure.

^{1}H NMR (300 MHz, $CDCl_3$) δ 7.32 – 7.22 (m, 5H), 5.98 – 5.87 (m, 1H), 5.30 - 5.19 (m, 2H), 4.86 -4.74 (br, 1H), 3.98 – 3.88 (m, 3H), 3.36 (m, 2H), 2.80 (dd, 2H), 1.44 (s, 9H).

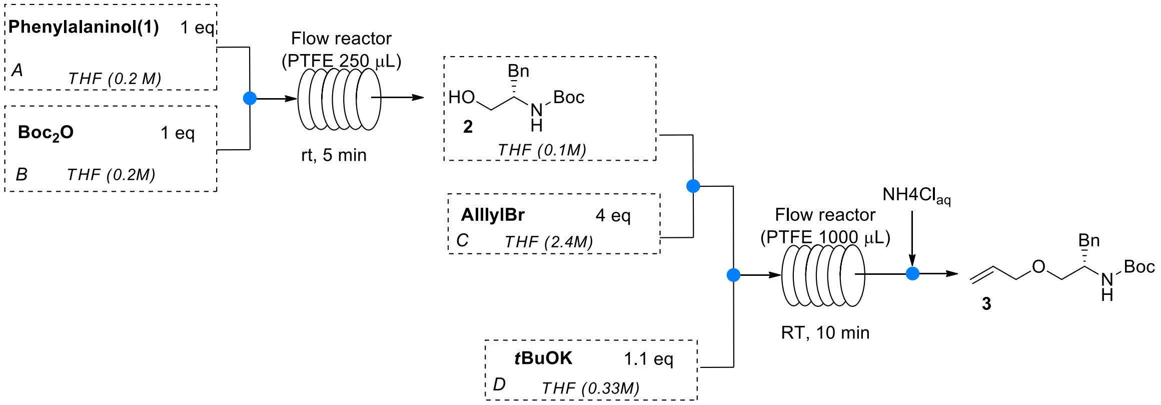

Two 2.5 mL Hamilton gastight syringes, containing *A* compound **1** (1 eq.) in 2 mL of THF (0.2 M), and *B* Boc_2O (1 eq.) in 2 mL of THF were connected by a PEEK tee junction to a 250 µL PTFE coil reactor. Both syringes fed the solutions ad 25 µL/min, giving a residence time of 5 minutes. The outcome of the reactor was connected to another tee junction, fed by a 1 mL Hamilton gastight syringe *C*, containing allylbromide (0.8 mL of 2.4 M solution in THF, 4 equivalents) feeding at 10 µL/min. The outcome of this second tee was connected to another tee junction, fed by a 5 mL Hamilton gastight syringe, *D*, containing *t*BuOK (1.6 mL of a 0.33 M solution in THF, 1.1 equivalents) with a flow rate of 20 µL/min. The outcome of this third tee was connected to a 1000 µL PTFE coil reactor, with a total flow rate of 80 µL/min, and a

subsequent residence time of 12.5 minutes. The outcome of the reactor was collected into a vial containing saturated NH_4Cl. The organic phase was separated, dried over sodium sulphate and evaporated under vacuo, to give product **3** with a 20% conversion, evaluated by ^{1}H NMR, and confirmed as isolated yield.

Analytical data are in agreement with product obtained in batch procedure.

In flow synthesis of <u>(*S*)-1-(allyloxy)-3-phenylpropan-2-amine</u> **4**

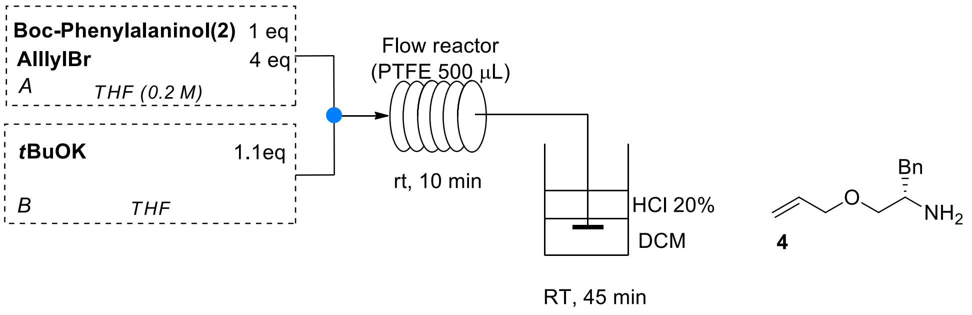

Two 2.5 mL Hamilton gastight syringes, containing *A* compound **2** (1 eq.) in 2 mL of THF (0.1 M), and 4 eq. of allylbromide, and *B* *t*BuOK (1.1 eq.) in 2 mL of THF were connected by a PEEK tee junction to a 500 µL PTFE coil reactor. Both syringes fed the solutions ad 25 µL/min, giving a residence time of 10 minutes. The outcome of the reactor was collected into a vial containing 10% HCl, were it was stirred for further 40 min. Then 1N NaOH was added until basic pH was reached, the organic phase was separated, dried over sodium sulphate and evaporated under vacuo, to give product **4** with a 75% conversion, evaluated by ^{1}H NMR, and confirmed as isolated yield.

Analytical data are in agreement with product obtained in batch procedure.

1H NMR (300 MHz, $CDCl_3$) δ 7.31 – 7.06 (m, 5H), 5.98 – 5.72 (m, 1H), 5.31 – 5.04 (m, 2H), 3.92 (d, J = 5.6 Hz, 2H), 3.44 – 3.29 (m, 1H), 3.28 – 3.09 (m, 2H), 2.72 (dd, J = 13.3, 4.9 Hz, 1H), 2.56 – 2.41 (m, 1H).

In flow synthesis of <u>(*S*,*Z*)-ethyl 3-((1-(allyloxy)-3-phenylpropan-2-yl)amino)-4,4,4-trifluorobut-2-enoate</u>

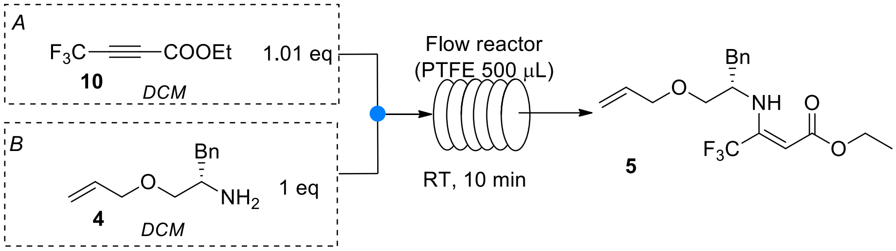

Two 2.5 mL Hamilton gastight syringes, containing *A* compound **10** (2 mL of a 0.8 M solution in CH_2Cl_2, 1.03 equivalents) and *B* compound **4** (2 mL of a 0.8 M solution in CH_2Cl_2, 1 equivalent) were connected by a PEEK tee junction to a 500 µL PTFE coil reactor. Both syringes fed the solutions ad 25 µL/min, giving a residence time of 10 minutes. The outcome of the reactor was collected in a vial cooled to -78°C, diluted with $CDCl_3$ (without removing CH_2Cl_2 to avoid the proceeding of the reaction during evaporation) and subjected to ^{19}F NMR, to evaluate the conversion of the product. The desired enamine was formed with an E/Z ratio of 7:3, evaluated by ^{19}F NMR. After purification, performed by column chromatography to confirm the isolated yield, the Z isomer converted into the E isomer. Analytical data are in agreement with product obtained in batch procedure.

^{1}H NMR (300 MHz, $CDCl_3$) δ 8.36 (d, *J* = 10.5 Hz, 1H), 7.17 (dd, *J* = 15.5, 12.7 Hz, 5H), 5.97 – 5.72 (m, 1H), 5.16 (dd, *J* = 31.3, 13.8 Hz, 2H), 4.98 (s, 1H), 4.09 (d, *J* = 7.1 Hz, 2H), 4.01 – 3.88 (m, 2H), 3.72 (s, 1H), 3.33 – 3.20 (m, 2H), 2.84 (dd, *J* = 32.6, 7.0 Hz, 2H), 1.25 – 1.15 (m, 3H).

^{19}F NMR (282 MHz, $CDCl_3$) δ -66.12 (s) (E isomer); -65.23 (s) (Z isomer).

In flow synthesis of Ethyl 3-(((*S*)-1-(allyloxy)-3-phenylpropan-2-yl)amino)-4,4,4-trifluorobutanoate **6**

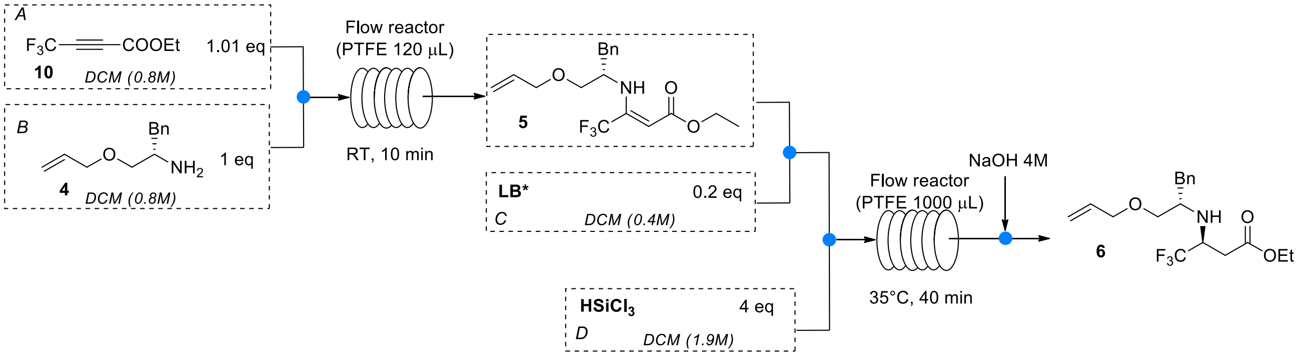

Two 2.5 mL Hamilton gastight syringes, containing *A* compound **10** (2 mL of a 0.8 M solution in CH_2Cl_2, 1.03 equivalents) and *B* compound **4** (2 mL of a 0.8 M solution in CH_2Cl_2, 1 equivalent) were connected by a PEEK tee junction to a 120 µL PTFE coil reactor. Both syringes fed the solutions ad 6 µL/min, giving a residence time of 10 minutes. The outcome of the reactor was connected to another tee junction, fed by a 1 mL Hamilton gastight syringe, *C*, containing **cat. ent-II** (0.8 mL of 0.4 M solution in CH_2Cl_2, 0.2 equivalents) feeding at 2.5 µL/min. The outcome of this second tee was connected to another tee junction, fed by a 5 mL Hamilton gastight syringe, *D*, containing $HSiCl_3$ (3.3 mL of a 1.9 M solution in CH_2Cl_2, 4 equivalents) with a flow rate of 10 µL/min. The outcome of this third tee was connected to a 1000 µL PTFE coil reactor, heated at 35°C, with a total flow rate of 24.5 µL/min, and a subsequent residence time of 40 minutes. The outcome of the reactor was collected into a NaOH 4 M solution at 0°C. After the first 2 volumes were discharged, steady state conditions were reached. The conversion of **6,** evaluated by ^{1}H NMR, was reported as an average of three reactors volume, separately collected. Reactors volumes were reunited and purified by column chromatography to confirm the conversion as isolated yield.

Analytical data are in agreement with product obtained in batch procedure.

^{1}H NMR (300 MHz, $CDCl_3$) δ 7.31-7.10 (m, 5H), 5.98 – 5.84 (m, 1H), 5.29 - 5.16 (m, 2H), 4.18 (q, 2H), 3.95 (d, *J* = 7.1 Hz, 2H), 3.80 (br s, 1H), 3.34 – 3.20 (m, 3H), 2.78 – 2.74 (m, 2H), 2.68 (dd, 2H), 2.45 (dd, 2H), 1.29 (t, 3H).

Pictures of Flow reactors set up

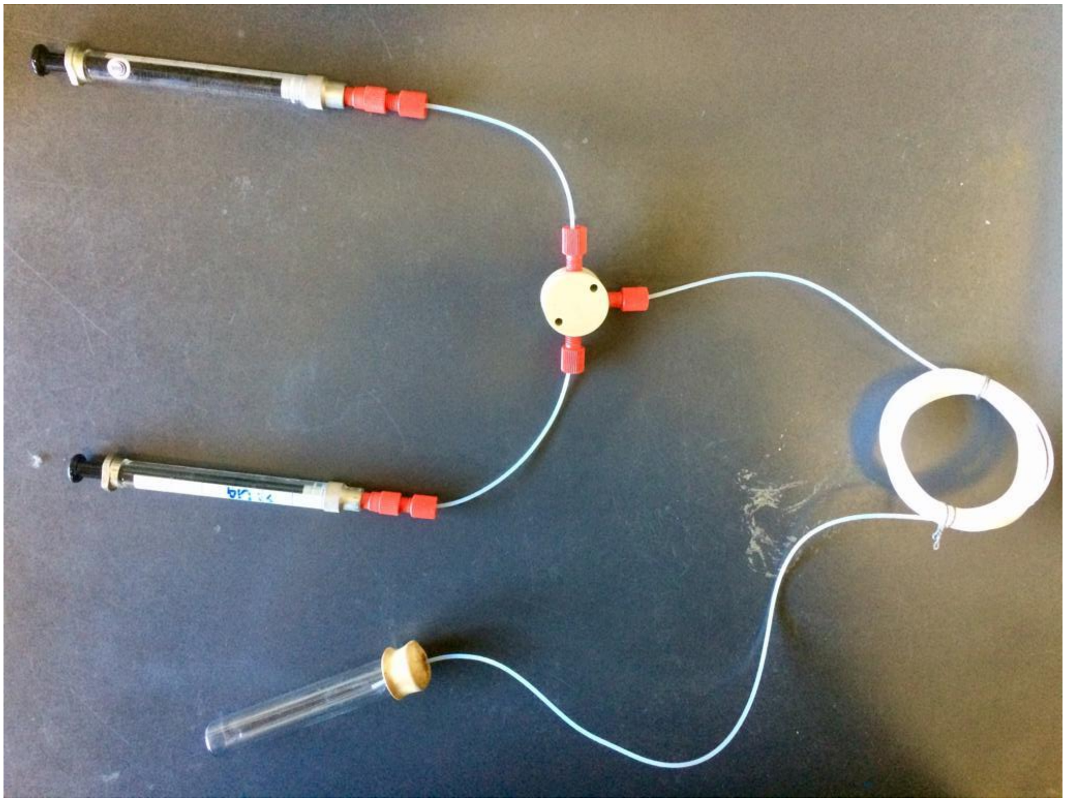

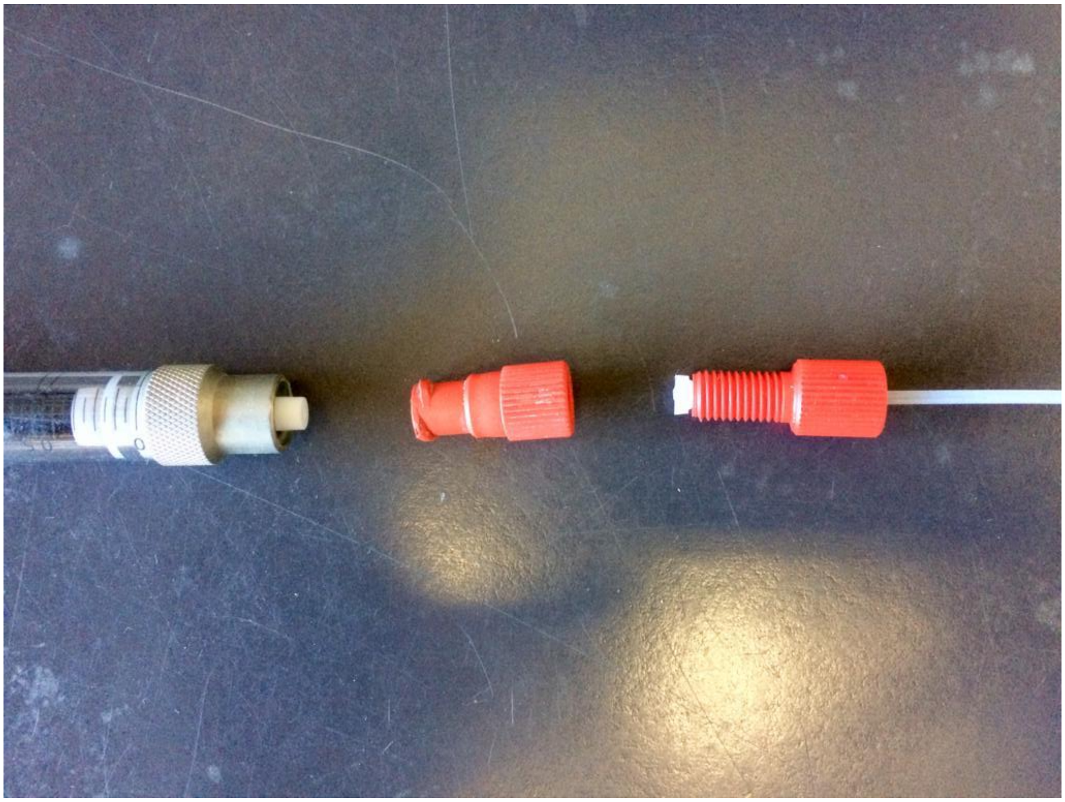

Supplement: Supplementary file 1 [file molecules-24-02260-s001.pdf]
